# Modelling of Hydrophilic Interaction Liquid Chromatography Stationary Phases Using Chemometric Approaches

**DOI:** 10.3390/metabo7040054

**Published:** 2017-10-24

**Authors:** Meritxell Navarro-Reig, Elena Ortiz-Villanueva, Romà Tauler, Joaquim Jaumot

**Affiliations:** Department of Environmental Chemistry, IDAEA-CSIC, Jordi Girona 18-26, 08034 Barcelona, Spain; meritxell.navarro@idaea.csic.es (M.N.-R.); elena.ortiz@idaea.csic.es (E.O.-V.); rtaqam@idaea.csic.es (R.T.)

**Keywords:** hydrophilic interaction liquid chromatography (HILIC), non-targeted metabolomics, stationary phase, chemometrics

## Abstract

Metabolomics is a powerful and widely used approach that aims to screen endogenous small molecules (metabolites) of different families present in biological samples. The large variety of compounds to be determined and their wide diversity of physical and chemical properties have promoted the development of different types of hydrophilic interaction liquid chromatography (HILIC) stationary phases. However, the selection of the most suitable HILIC stationary phase is not straightforward. In this work, four different HILIC stationary phases have been compared to evaluate their potential application for the analysis of a complex mixture of metabolites, a situation similar to that found in non-targeted metabolomics studies. The obtained chromatographic data were analyzed by different chemometric methods to explore the behavior of the considered stationary phases. ANOVA-simultaneous component analysis (ASCA), principal component analysis (PCA) and partial least squares regression (PLS) were used to explore the experimental factors affecting the stationary phase performance, the main similarities and differences among chromatographic conditions used (stationary phase and pH) and the molecular descriptors most useful to understand the behavior of each stationary phase.

## 1. Introduction

Over the past decade, metabolomics has received considerable attention from the scientific community. Metabolomics aims to screen endogenous small molecules (metabolites) present in biological samples, providing a direct measure of the phenotypic state of an organism [1,2,3]. There are two main metabolomic strategies: targeted and non-targeted. The targeted approach is focused on the investigation of a specific metabolic pathway and, therefore, in the analysis of a reduced and known set of compounds. In contrast, non-targeted metabolomics aims to screen the entire metabolite content of biological samples containing compounds with different physical and chemical properties [4,5]. 

Due to its high-resolution power, sensitivity and accuracy of *m/z* detection, liquid chromatography coupled with high-resolution mass spectrometry (LC-HRMS) has become the analytical platform most used in metabolomics studies. Reversed phased liquid chromatography (RPLC) is useful for the separation of the more hydrophobic compounds, such as lipids. However, RPLC is not recommended for the analysis of some of the most usual metabolite families characterized as being polar and hydrophilic compounds [2,6,7,8,9]. In the analysis of polar compounds, and in particular in the metabolomics field, hydrophilic interaction liquid chromatography (HILIC) has become a valuable alternative to RPLC, due to its ability to separate these more hydrophilic compounds [10,11]. Different types of HILIC stationary phases, such as amide, amine, mixed-mode diol and zwitterionic, have been employed previously in metabolomics studies [11,12,13]. This variety of HILIC stationary phases allows the separation of metabolites of different properties. Nevertheless, this diversity also makes the selection of the most suitable HILIC stationary phase for a particular study more challenging [10,11,13]. Moreover, the retention mechanism in HILIC has been demonstrated to be more complex than in RPLC mode [14]. In HILIC mode, the separation is primarily achieved due to the partition of analytes between the mobile phase and the hydrophilic layer adsorbed at the surface of the stationary phase. Also, it is proposed that other electrostatic interactions, such as ion exchange interactions, may also contribute to the retention mechanisms. These electrostatic interactions vary among the different types of HILIC stationary phases, making their comparison and selection more difficult [15,16].

Chemometric tools can help in addressing the challenge of finding the most suitable HILIC stationary phase for the analysis of a complex mixture of polar compounds [17]. On the one hand, multivariate data exploratory methods, such as principal component analysis (PCA) [18], can be used to investigate the behavior of HILIC stationary phases and understanding retention mechanisms. Other multivariate statistical methods, like ANOVA-simultaneous component analysis (ASCA) [19], can help evaluating the statistical significance of the experimental factors involved in a non-targeted metabolomics study, such as when different HILIC stationary phases and mobile phase conditions are assessed. On the other hand, building models linking the physicochemical properties of compounds (molecular descriptors, MDs) and their chromatographic behavior (retention factors) could help to get a different insight into the HILIC stationary phase performance [11,20,21,22,23,24,25,26,27,28,29]. In addition, these models could also be used to predict the behavior of a chromatographic system and, therefore, can be used to predict the chromatographic retention of unknown compounds and, in some cases, provide additional information to support their identification [11,23,24,30]. 

In a previous work, different types of HILIC stationary phases have already been evaluated for metabolomics studies [17]. However, in that preliminary work, only 12 metabolites were considered, which simplified the analysis considerably. Moreover, the detection was performed using diode array detector (DAD), which is not the standard in complex studies in the metabolomics field. In contrast, here, a mixture of 54 metabolites is analyzed using LC-HRMS. Four different types of HILIC stationary phases commonly used in metabolomics research have been evaluated under various experimental conditions using different chemometric tools for their application to non-targeted studies. First, PCA and ASCA were applied to explore the general behavior of the considered stationary phases and to evaluate the statistical significance of the three experimental factors discussed in this work (stationary phase, pH and ionic strength). Then, partial least squares regression (PLS) models based on MDs computed from molecular structures of different metabolite families were calculated for evaluating the chromatographic behavior of the four HILIC stationary phases considering the retention factor of the analyzed metabolites. 

## 2. Results and Discussion

### 2.1. Determination of Retention Factors

A mixture of 54 metabolites from different families (see Table 1) was analyzed using four different stationary phases (Ethylene Bridged Hybrid (BEH) amide, amide, zwitterionic and mixed-mode diol), working with mobile phase at three pH values (acid, moderately acid and neutral) and two ionic strengths (low and high). Each chromatographic condition was injected twice, giving a total of 48 chromatographic runs. The regions of interest (ROI) strategy was used to arrange the 48 chromatographic runs in LC-MS data matrices. Then, each matrix was evaluated to automatically find the *m/z* value of each metabolite and provide their retention time in each chromatogram. Lastly, retention factor (k) of each metabolite in each chromatographic run was calculated using equation 1 (see Materials and methods section for more details). 

**Figure 1:**
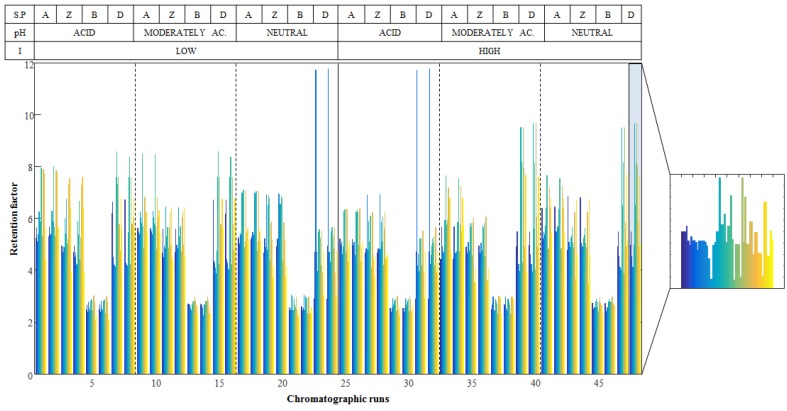
Retention factors for the 54 metabolites in the 48 chromatographic runs (matrix **D**). The table on the top shows the chromatographic conditions of each sample: (A) indicates the chromatographic runs perfumed using amide, (Z) zwitterionic, (B) BEH amide and (D) mixed-mode diol stationary phases. As an example, a zoomed view of chromatographic run 48 is depicted.

Finally, a matrix, **D**, containing the retention factors of metabolites at each chromatographic condition was built up. This matrix had a number of rows equal to the number of chromatographic runs performed (48 runs) and a number of columns equal to the number of analyzed metabolites (54 compounds). Figure 1 shows the obtained retention factors for the 54 metabolites in the 48 chromatographic runs. The visual inspection of the obtained retention factors already allowed the differentiation of the evaluated chromatographic conditions. For instance, the retention factors of all metabolites in BEH amide are shorter than in the rest of the stationary phases. However, direct evaluation of the HILIC stationary phases behavior is not straightforward. For this reason, matrix **D** was first evaluated using two chemometric exploratory methods, PCA and ASCA, to get a deeper insight into the effects of experimental factors on the retention behavior of metabolites. 

### 2.2. Evaluation of HILIC Stationary Phases Behavior

**Figure 2:**
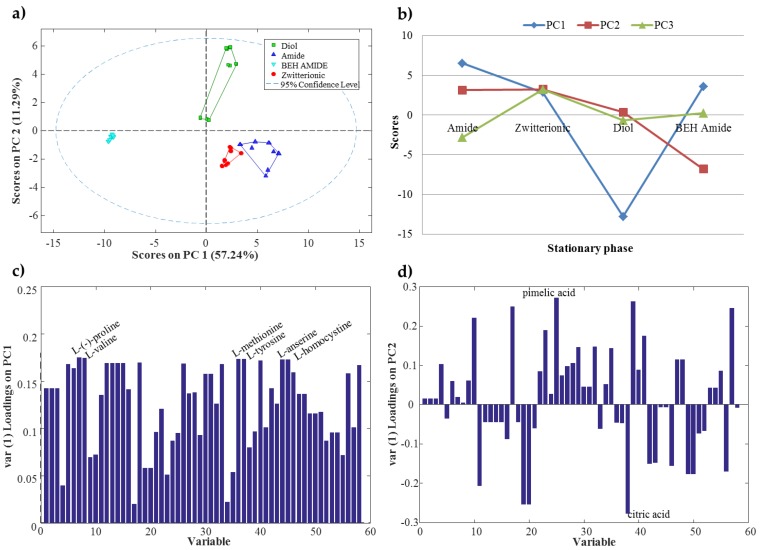
(**a**) Principal component analysis (PCA) scores plot of samples classified according to the stationary phase used in the chromatographic system; (**b**) ANOVA simultaneous component analysis (ASCA) principal component scores for the stationary phase factor; (**c**) ASCA PC1 loadings for the stationary phase factor; (**d**) ASCA PC2 loadings for the stationary phase factor.

PCA was applied to matrix **D** in order to explore the behavior of the chromatographic systems studied in this work (four different stationary phases and mobile phase at three different pH conditions and two different ionic strengths). PCA results indicated that stationary phase was the most critical factor to be considered. In Figure 2a, the PCA scores plot shows that samples analyzed with the four stationary phases were differentiated. Moreover, PC1 distinguished BEH amide stationary phase (XBridge^TM^ Amide) samples (cyan triangles in Figure 2a), which appeared on the left side of PC1, from the rest of the samples. Additionally, PC2 distinguished mixed-mode diol stationary phase (Acclaim^TM^ Mixed-Mode HILIC-1) samples (green squares in Figure 2a), with large positive PC2 scores values, from the rest of the samples. Amide (TSK-Gel Amide-80) and zwitterionic (ZIC-HILIC) stationary phases showed a similar behavior since their samples appeared close to each other in the scores plot. Furthermore, samples analyzed with the three different pH values were also clearly distinguished (see Figure S1 in Supplementary Information), whereas, samples analyzed at different ionic strengths (low and high) could not be differentiated by the PCA scores plot. These results were consistent with the results obtained in the previous authors’ work about HILIC stationary phases [17]. 

Statistical significance of the three experimental factors considered in this work (stationary phase, pH and ionic strength) and their interactions were assessed by applying ASCA to matrix **D**. Results showed that both stationary phase and pH had statistically significant effects (*p*-value of 0.0001). On the contrary, the ionic strength effects were not significant (*p*-value of 0.2). Moreover, the interaction of three factors (stationary phase × pH, stationary phase × ionic strength and pH × ionic strength) was also found to be statically significant (*p*-value of 0.0001). Hence, from the combination of ASCA and PCA results, the two most relevant factors were defined as the stationary phase and the pH of the aqueous solvent. 

Figure 2b shows the ASCA principal component scores (at the mean level) for each HILIC stationary phase. In this scores plot, some trends could be observed. For example, PC1 distinguished the mixed-mode diol stationary phase with a large negative scores value, whereas the two amides and the zwitterionic stationary phases showed a similar positive PC1 scores value. PC2 also differentiated the BEH amide stationary phase with a negative scores value. In contrast, amide, zwitterionic and mixed-mode diol stationary phases had a similar positive PC2 scores value. Finally, it should be mentioned that, as observed in PCA results, amide and zwitterionic stationary phases had similar PC1 and PC2 scores values. Therefore, these two stationary phases showed a similar behavior. 

ASCA loadings were useful to know which variables (metabolite retention factors) were the most important to distinguish the stationary phases. Figure 2c,d show the ASCA loadings plot for PC1 and PC2, respectively. For instance, six amino acids (l-(−)-proline, l-valine, l-methionine, l-tyrosine, l-homocysteine and l-anserine) showed a higher loadings value in PC1 (Figure 2c). Consequently, these amino acids were useful to distinguish mixed-mode diol stationary phase from the other three (amide, BEH amide and zwitterionic). In the case of PC2 (Figure 2d), pimelic and citric organic acids showed the highest loadings values. Therefore, these two organic acids appeared as important to differentiate BEH amide from the rest of the stationary phases. 

In general, PCA and ASCA results coincided showing that the most important factors were the HILIC stationary phase and the pH of the aqueous solvent. In addition, some facts related to the stationary phase behavior can be highlighted. For instance, the zwitterionic stationary phase showed an intermediate behavior between the two amide stationary phases. 

The next step in this work was to find relationships between the observed chromatographic retention observed and the physicochemical properties of metabolites using their molecular descriptors. In addition, since PCA and ASCA results showed that the ionic strength of the mobile phase was not a significant factor in metabolomics studies, these new PLS models were only assessed considering chromatographic runs done at low ionic strength.

### 2.3. Exploratory Relationship between Physicochemical Properties and HILIC Chromatographic Retention 

PLS models were independently built for the 12 chromatographic studied systems. They were obtained by the combination of the four stationary phases (BEH amide, amide, zwitterionic and mixed-mode diol) and of the three mobile phases at different pH values (acid, moderately acid and neutral). These models were generated using the experimental retention factors of the 54 metabolites contained in the mixture (a **y** vector for each condition) and their corresponding molecular descriptors (MDs) organized in an **X** matrix. A preliminary selection step was performed over the whole set of MDs available from PCLIENT to reduce the total number and finally consider 844 of them (see Materials and Methods section for more details). 

**Figure 3:**
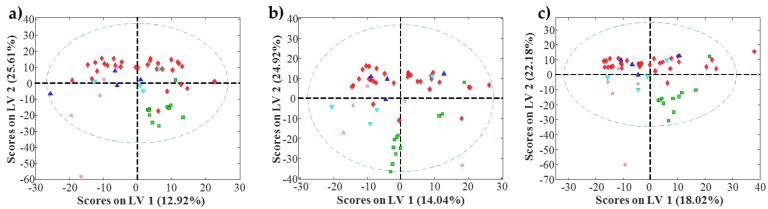
Partial Least Squares (PLS) scores plot for amide stationary phase. (**a**) Acid pH; (**b**) Moderately acid pH; (**c**) Neutral pH. Red diamonds are amino acids (♦), green squares are nucleosides (■), blue triangles are organic acids (▲), purple stars are sugars (★) and cyan triangles are others (▼).

PLS modelling of the retention factor obtained for each condition using metabolite molecular descriptors required a reduced number of latent variables (between 2 and 3) to explain most of the variance of each **y** vector (between 80% and 95%). These models did not show an accuracy enough to be used for the prediction of the retention factors of unknown compounds. However, as these PLS models explain a major part of the retention factor variance, the exploration of scores and loading plots can provide additional insight into the HILIC behavior. First, the analysis of scores plots could allow confirming the differentiation between groups of samples. More interestingly, the analysis of loadings plots could provide information regarding the molecular descriptors related to this differentiation, and could give additional information to known the main physicochemical properties involved in the HILIC retention mechanisms. As an example, Figure 3 shows the scores plots obtained for the amide stationary phase at the three studied pH values of the mobile phases. In these plots, some interesting trends can be observed. First, the differentiation between nucleosides and the rest of the metabolites present in the mixture. In the three cases, a clear group with all nucleosides is visible. Amino acids are the metabolite family with the most compounds in the mixture. These amino acids are spread along the first latent variable. However, in the three pH conditions, differentiation between two groups can be detected (Figure 3a). An inspection of metabolites forming these two groups allowed observing that the left group was composed of metabolites with a molecular weight lower than 130 Da, whereas the right group was composed of metabolites with a molecular weight larger than 130 Da. Regarding the other families of metabolites present in the mixture (i.e., sugars or organic acids), metabolites were grouped but they were overlapping with amino acids. Similar trends can be observed when considering the other chromatographic conditions.

The evaluation of which MDs allowed obtaining this retention factor modelling is more appealing. The identification of MDs can be performed using the variable importance on projection (VIP) scores obtained for each PLS model as a feature selection tool allowing to identify which variables (MDs) were the most descriptive of every chromatographic system studied in this work. Table 2 shows the twenty most relevant MDs for each PLS model and their VIP scores values. MDs that appeared to be important in more than one chromatographic system are shown in bold letters. The most repeated MDs values present in Table 2 were the 3D-MoRSE values like the molecular representation of structures based on electronic diffraction, Mor02p, Mor10p, Mor15p, Mor08u, Mor19u, Mor24u, Mor31u, Mor01m, Mor06m, Mor10m, Mor 10v, Mor15v, Mor08e, Mor24e and Mor31e. All of them are geometrical descriptors that codify the 3D molecular structure [31,32,33,34]. Some other geometrical descriptors (HTcxp, RTe and RTm) also seemed relevant. Geometrical descriptors are calculated from the coordinates of the molecule atoms, interatomic distances and distances from a specific origin. They are geometrical descriptors of the molecular size, shape, symmetry and atom distribution [33,34]. Moreover, these descriptors are weighted by ionization potential, electronegativity, polarizability and molecular mass [11]. Different topological descriptors (J, Lop, MWC03, MWC04, EEig10r, ESpm01d, ESpm07d, ESpm09d, ESpm13d, ESpm14d and ESpm14r) were also highlighted as relevant for the modelling of the studied chromatographic systems. These topological descriptors are numerical quantifiers of molecular topology that are sensitive to one or more structural properties, such as size, shape, symmetry or branching, and can also include chemical information about atom type and bond multiplicity [34,35,36,37]. Autocorrelation descriptors (GATS2e, GATS2p, GATS2m, GATS2v, MATS4e, MATS4p, MATS4m, MATS4v, MATS7v, RTm and ATS3m) also appeared to be significant in almost all the studied systems. These descriptors encode both molecular structure and physicochemical properties of a molecule (molecular mass, van der Waals volume, electronegativity or polarizability) [34,38,39]. Constitutional descriptors and molecular properties, like the number of double bonds (nDB) and the number of ratable bonds (RBN), the unsaturation index (Ui) and the octanol-water partition coefficient (ALOGP and MLOGP) also were relevant in the obtained PLS models. Finally, MDs related to connectivity had significant VIP values for the studied systems. These MDs are called BCUT (Burden eigenvalue descriptors) descriptors (BELe3) [34,40] and Randic connectivity indexes (VRv2 and VRp2) [34,41]. In this case, the selection of different descriptors related to the Sanderson electronegativity can be mentioned giving a preliminary insight into the interaction mechanism between metabolites and stationary phases. 

**Figure 4:**
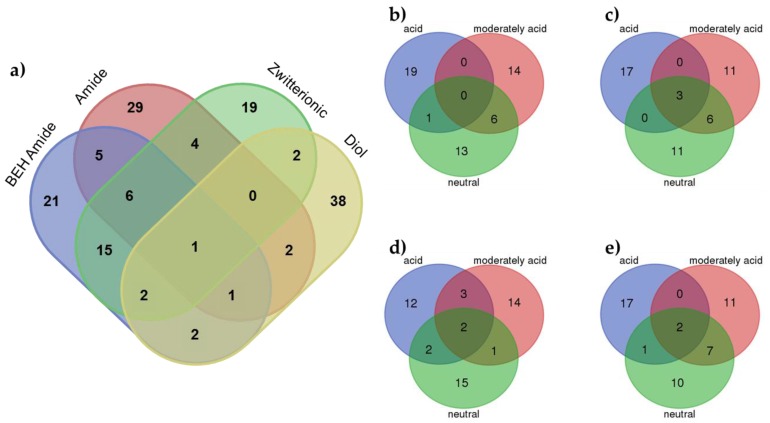
Venn diagrams of the VIPs MDs at (**a**) each stationary phase considering all pH values; (**b**) BEH Amide at each pH condition; (**c**) Amide at each pH condition; (**d**) Zwitterionic at each pH condition and (**e**) Diol at each pH condition.

A deep analysis of the identified molecular descriptors and their relationships with the significant experimental factors (stationary phase and pH value) allowed finding some interesting trends. Figure 4a shows a Venn diagram showing the MDs for each stationary phase considering all pH values. In this plot, the different behavior of the diol stationary phase can be observed. Most MDs (38) were unique, and only some of them appeared as relevant to other stationary phases. This difference in the behavior of the mixed-mode diol stationary phase can be explained by the mixed chemistry of the surface with a hydrophobic alkyl chain with a diol group. These dual properties allow the use of this stationary phase for both RP and HILIC separations but, from our results, modelling using a PLS model approach was more difficult. In addition, despite the ionic strength factor not being significant (using ASCA), the mixed-mode diol stationary phase seemed to be more affected than the other stationary phases, which could also be related to worse modelling. When considering amide, BEH amide and zwitterionic stationary phases, more similarities in the identified MDs were observed. In addition, from these results, and in accordance with PCA, the zwitterionic stationary phase seemed to have an intermediate behavior between the two amide stationary phases. Finally, evaluation of the MDs identified for the different stationary phases at different pH values showed that most of MDs were unique for a particular condition. However, more similarities can be observed between the moderately acid and neutral pH values (especially in the case of the diol stationary column, confirming its different behavior).

## 3. Materials and Methods

### 3.1. Chemicals and Reagents

Acetic acid (≥95.0%), formic acid (≥95.0%), ammonia (25%), LC-MS water and Acetonitrile (ACN, LC-MS grade) were obtained from Merck (Darmstadt, Germany). Ammonium acetate (≥99.0%) was supplied by Sigma-Aldrich (St. Louis, MO, USA). 

A mixture of 54 metabolites was used to evaluate the HILIC stationary phases behavior. Table 1 shows the metabolites contained in the analyzed mixture. All standards were purchased from Sigma-Aldrich (St. Louis, MO, USA). Nucleosides and amino acids were from two mix solutions provided by Sigma-Aldrich (St. Louis, MO, USA). A summary of the polarity of the analyzed metabolites is shown in Table S1 in the supplementary information. 

Standard stock solutions (1000 μg mL^−1^) of metabolite mixture were prepared by dissolving an appropriate amount of each metabolite in water and stored at −20 °C until their use. Working standard solutions (20 μg mL^−1^) were prepared by diluting the stock solution in ACN:H2O (1:1). 

### 3.2. Instrumentation

The metabolite mixture was analyzed using an Acquity UHPLC system (Waters, Milford, MA, USA) for the chromatographic separation, equipped with a quaternary pump, an autosampler and a column oven. The mass spectrometer was a triple quadrupole detector (TQD, Waters, Milford, MA, USA) equipped with an electrospray (ESI) ionization source in negative and positive modes. The mass acquisition range was set to 90–1000 *m/z*. 

Four different HILIC stationary phases (BEH amide, amide, zwitterionic and mixed-mode diol) were evaluated (properties summarized in Table 3). The elution gradient was performed using solvent A (acetonitrile) and solvent B (ammonium acetate buffer solution). Chromatographic conditions used for each column are also detailed in Table 3. In order to reproduce the most used chromatographic conditions in metabolomics studies, the experiments were performed using solvent B at three different pH values: acidic (3.0 adjusted with formic acid), moderately acidic (5.5 adjusted with acetic acid) and neutral (7.0 adjusted with ammonia). The pH measurements were performed at 25 °C using an Orion Star A111 pH meter (Thermo Scientific, Waltham, MA, USA), before the additions of the organic solvent. Moreover, two ionic strengths in the aqueous phase were also compared: low (5.0 mM) and high (25 mM). 

The metabolite mixture was analyzed with the four stationary phases working with solvent B at the three pH values and the two ionic strengths. Each condition was injected twice giving an experimental design with a total number of 48 chromatographic runs.

### 3.3. Data Analysis

#### 3.3.1. Retention Factor Determination

**Figure 5:**
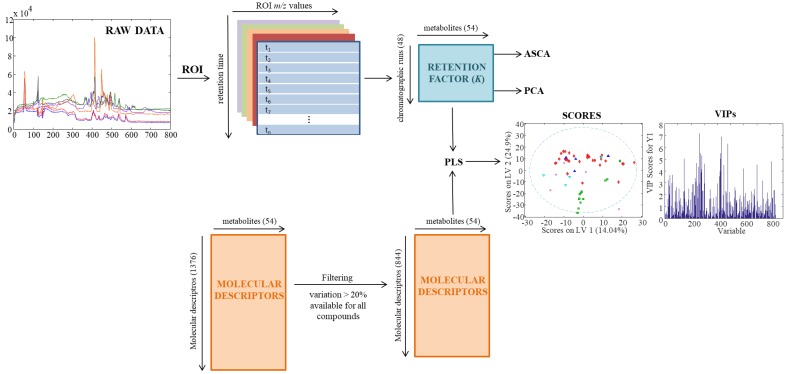
Scheme of data analysis strategy.

Figure 5 shows a complete picture of the data analysis strategy from the raw MS data to the chemometric modelling. First, raw chromatographic data files (in .raw format) were converted to the standard CDF format by Databridge function of MassLynx^TM^ v 4.1 software (Waters, Milford, MA, USA). Then, these data files were imported into the MATLAB environment (Release 2015b, The Mathworks Inc., Natick, MA, USA) by using *mzcdfread* and *mzcdf2peak* functions of the MATLAB Bioinformatics Toolbox (4.3.1.version). LC-MS data were then arranged and aligned according to their m/z in a data matrix, containing retention times in the rows and selected *m/z* values in the columns. Here, this data matrix was built up using the previously proposed regions of interest (ROI) strategy [42,43]. The ROI approach selects the most relevant mass traces, which are those *m/z* values whose intensity signals are higher than a fixed signal-to-noise ratio threshold and appear a number of times consecutively in the time dimension. These mass traces are searched among all the chromatographic and spectral data. The obtained vectors, containing the intensity of the found ROIs at each time point, are reorganized into a matrix grouping ROIs among all the retention times. The final *m/z* values of each ROI are calculated as the mean of all *m*/*z* values obtained for that particular ROI. In this work, the parameters for the implementation of this ROI approach are the signal-to-noise ratio threshold (set at 0.1% of the maximum MS signal intensity), the mass accuracy of the mass spectrometer (set at 0.5 Da/e for the TQD MS analyzer used in this work) and the minimum number of consecutive retention times to be considered as a chromatographic peak (set at 25). More details on how this strategy works are given in previous works [1]. Finally, an ROI matrix was obtained for positive and negative ionization modes for each of the 72 chromatographic runs of the present study.

Every ROI matrix corresponding to each chromatographic run was then evaluated to automatically find the *m/z* value of each metabolite and provide their retention time in each chromatogram. Lastly, retention factor (*k*) of each metabolite in each chromatographic run was calculated using their retention times (*t*_R_) and the dead time (*t*_0_, theoretically obtained from the dead volume) as follows:

(1) $$k=\frac{t_{R}-t_{0}}{t_{0}}$$

#### 3.3.2. Molecular Descriptors Determination

Canonical SMILES representations for the standard metabolites were retrieved from PubChem [44] and HMDB [45] databases. These SMILES were input into the PCLIENT software to calculate molecular descriptors (MDs). PCLIENT software can calculate more than 3000 MDs that are divided into 25 logical blocks. Here 1376 MDs were calculated including constitutional, topological, geometrical, electrostatic, physical, shape, and quantum chemical descriptors. Details of MDs calculation can be found in the Handbook of Molecular Descriptors [34]. PCLIENT software is available online at http://www.vcclab.org. When 3D atom coordinates were needed for parameter calculation, they were obtained using CORINA software (Molecular Networks GmbH, Nürnberg, Germany).

To reduce the number of MDs descriptors with a percentage of variation (calculated dividing the standard deviation of the MD values by their mean) lower than 20% were excluded. Also, those descriptors not available for all compounds were removed. After this reduction, 844 MDs were obtained for further analysis. 

#### 3.3.3. Evaluation of HILIC Chromatographic Performance

The retention factors of the 54 metabolites in each HILIC stationary phase at different chromatographic conditions were used to investigate the behavior of the chromatographic systems studied in this work by explorative chemometric methods. 

The behaviors of the chromatographic systems studied in this work were evaluated using the retention factors of the 54 metabolites. The retention factor data matrix **D** (containing the retention factor of 54 metabolites at the 48 chromatographic runs) was evaluated using diverse chemometrics exploratory methods: principal component analysis (PCA) [18], ANOVA-simultaneous component analysis (ASCA) [19] and partial least squares regression (PLS). 

PCA [18] compresses the information of the original variables into a smaller number of uncorrelated variables known as principal components [18]. In this work, PCA was applied to evaluate the relationships between the experimental conditions studied: stationary phase, pH and ionic strength. Therefore, matrix **D** was analyzed and information about the chromatographic runs and metabolite distribution were obtained in scores and loadings, respectively. 

ASCA [19] is a multivariate analysis of variance method that combines the capacity of ANOVA to separate variance sources with the advantages of simultaneous component analysis (SCA, a generalization of PCA for the situation where the same variables have been measured in multiple conditions) [46]. In this work, ASCA was applied to statistically assess the significance of experimental factors in the experimental design: stationary phase, pH and ionic strength. ASCA was performed on a well-balanced experimental design, and 10,000 permutations were used for the permutation test [47]. More details about the ASCA method can be found in the work of Smilde [19] and Jansen [48]. Data were autoscaled prior to applying PCA and centered before applying ASCA.

Finally, PLS regression was used to explore the relationships between obtained retention factors for each chromatographic condition and molecular descriptors (MDs). PLS [49,50,51] is a multivariate linear regression model used to find correlation models between predictor variables (**X** data matrix) and response values to be predicted (**y** vector). In this work, PLS is used as a regression analysis method to build a model to link the determined retention factor of metabolites (arranged in a vector **y**) using their MDs (arranged in matrix **X**) and to investigate the most influential MDs in the regression. In this work, the optimum number of latent variables for each model was selected using leave-one-out cross-validation. 

PLS also provides information about the most relevant variables for achieving the retention factors modelling. For instance, variables importance in projection (VIP) scores can be used for that purpose [51]. According to the common use, variables with a VIP score greater than 1 were important [52]. In this work, these VIP variables corresponded to those MDs that allowed a better description of the retention factor for each considered metabolite.

PCA, ASCA, and PLS were performed using PLS Toolbox 8.0 (Eigenvector Research Inc., Wenatchee, WA, USA) working under MATLAB (The Mathworks, Natick, MA, USA). 

## 4. Conclusions

Results obtained in the assessment of the behavior of HILIC chromatographic stationary phases by means of a variety of chemometric methods showed that the two most important factors to be considered in metabolic studies are the stationary phase and the pH of the aqueous solvent. Moreover, BEH amide and mixed-mode diol stationary phases behaved rather differently compared to amide and zwitterionic phases, which performed similarly. ASCA loadings were useful to know which metabolites were the most important to distinguish the stationary phases. Amino acids appeared to be useful to distinguish the mixed-mode diol stationary phase, while organic acids seemed to distinguished BEH amide from the rest of the stationary phases. In addition, exploratory PLS models allowed linking the retention of metabolites at different chromatographic conditions with molecular descriptors defining their physicochemical properties. Again, a similar behavior was observed for amide and zwitterionic stationary phases whereas the mixed-mode diol stationary phase showed a different performance. 

Finally, the obtained PLS models could be considered as a starting point in a more comprehensive work for modelling and prediction of chromatographic retention factors of metabolites in different HILIC stationary phases. However, building up these models requires bigger metabolite datasets with a larger number of compounds for each of the metabolite families. Moreover, efforts should be made in performing a comprehensive external validation of the models. Future work should address these issues. 

## Figures and Tables

**Table 1:** Metabolites contained in the analyzed mixture.

Metabolite Families					
Nucleosides	Amino Acids		Sugars	Organic Acids	Others
1-methyladenosine	1-methyl-l-histidine	l-citrulline	d(−)-ribose	citric acid	hypoxanthine
2′′-*O*-methylcytidine	3-methyl-l-histidine	l-glutamic acid	glucose	ketoglutaric acid	l-carnitine
2-thiocytidine	4-hydroxy-l-proline	l-histidine	trehalose	pimelic acid	serotonin
5-methylcytidine	5-hydroxylysine	l-homocystine	mannitol	succinic acid	tryptamine
cytidine	β-alanine	l-isoleucine	-	creatine	-
guanosine	creatinine	l-leucine	-	-	-
inosine	cysteine	l-methionine	-	-	-
pseudouridine	l-(−)-proline	l-ornithine	-	-	-
ribothymidine	l-(+)-arginine	l-serine	-	-	-
uridine	l-(+)-cystathionine	l-threonine	-	-	-
-	l-(+)-lysine	l-tryptophan	-	-	-
-	l-2-aminoadipic acid	l-valine	-	-	-
-	l-2-amino-n-butyric acid	taurine	-	-	-
-	l-alanine	sarcosine	-	-	-
-	l-anserine	l-aspartic acid	-	-	-
-	l-carnosine	-	-	-	-

**Table 2:** Variable importance on projection (VIP) scores of the twenty most important molecular descriptors (MD) for each chromatographic system.

**BEH Amide Acid**		**BEH Amide Moderately Acid**		**BEH Amide Neutral**		**Amide Acid**		**Amide Moderately Acid**		**Amide Neutral**	
**MD**	**VIP**	**MD**	**VIP**	**MD**	**VIP**	**MD**	**VIP**	**MD**	**VIP**	**MD**	**VIP**
**EEig10r**	13.4	**RTe**	9.62	**Mor24u**	8.80	G2p	12.3	Mor02e	8.27	**Mor31e**	13.8
**MWC03**	11.5	**BELe3**	7.92	**Mor24e**	8.62	G2m	11.5	ESpm02x	7.89	**Mor31u**	13.8
**Mor31e**	9.76	**BLTD48**	7.92	RDF080v	7.95	G2u	11.1	**Mor31u**	6.76	**Mor08u**	9.85
**GATS2v**	8.72	**BLTA96**	7.89	**GATS2e**	6.10	G2v	11.1	**Mor31e**	6.70	**Mor08e**	8.85
**GATS2p**	8.66	**Mor08u**	7.84	**BELe3**	5.64	G2e	11.0	**Mor10m**	6.61	EPS1	7.04
**Mor31u**	8.56	**Mor24u**	7.19	**BLTA96**	5.61	**Mor31e**	9.29	**ESpm09d**	5.87	**Mor06m**	6.08
**Mor02p**	8.50	**Lop**	6.69	**BLTD48**	5.61	BELe2	9.05	**GATS2e**	5.81	GATS6v	6.00
**GATS2m**	8.33	G3v	6.63	ESpm09x	5.00	**Mor02p**	8.74	**Mor06m**	5.74	**Mor15v**	5.79
ESpm05d	8.08	**Mor08e**	6.59	**ESpm14d**	4.980	**Mor31u**	8.45	RDF020p	5.54	Mor08p	5.64
**Mor06m**	8.04	G3p	6.57	Mor19e	4.87	BEHp1	7.32	CIC0	5.41	**Mor15p**	5.50
**MATS4m**	7.45	G3u	6.39	R4e	4.76	G1u	6.99	**Mor08u**	5.39	**Mor10m**	5.40
**MATS4e**	6.74	G3s	6.29	**TIC4**	4.73	G1e	6.64	WA	5.32	RDF080m	5.31
**MATS4v**	6.44	G3e	6.27	**Mor08u**	4.71	G1m	6.60	**Mor08e**	5.08	**GATS2v**	5.00
Mor16e	5.39	**Mor10p**	6.05	**TIC3**	4.59	G1p	6.48	R5e	4.98	**Mor10v**	4.64
GATS4v	5.35	**Mor24e**	6.02	AAC	4.56	G1v	6.45	**RTm**	4.80	**RTm**	4.54
**HVcpx**	5.33	G3m	5.91	IC0	4.56	**MWC03**	6.21	**Mor01m**	4.74	Mor08v	4.52
SP03	5.23	**Mor10v**	5.54	piID	4.49	BELp2	6.10	VDA	4.68	ESpm14x	4.48
Mor17e	5.16	RDF075m	5.39	ESpm02u	4.41	**Mor10m**	5.25	RDF070u	4.64	**RBN**	4.47
**MATS4p**	4.92	**VRp2**	5.37	**Mor19u**	4.41	**HVcpx**	5.22	**Mor19u**	4.30	**ALOGP**	4.32
**GATS2e**	4.71	**VRv2**	5.37	ESpm12r	4.41	**MATS4m**	5.15	**RBN**	4.27	**Mor19u**	4.32
**Zwitterionic Acid**		**Zwitterionic Moderately Acid**		**Zwitterionic Neutral**		**Diol Acid**		**Diol Moderately Acid**		**Diol Neutral**	
**MD**	**VIP**	**MD**	**VIP**	**MD**	**VIP**	**MD**	**VIP**	**MD**	**VIP**	**MD**	**VIP**
**MATS7v**	9.20	**EEig10r**	7.99	**MATS4m**	1.11	**ESpm09d**	1.50	G(O..O)	9.65	**Ui**	6.84
**Mor24u**	8.03	L3u	7.07	**MATS4e**	1.00	ESpm02r	1.42	IC3	8.52	**ESpm07d**	6.54
HTv	7.87	Mor18e	6.69	**MATS4v**	9.75	ESpm06d	1.12	**ATS3m**	7.82	**J**	6.49
**Mor24e**	7.42	**TIC4**	6.65	**Mor31e**	7.65	**ESpm13d**	7.21	T(O..O)	7.66	**ESpm01d**	6.41
BELe3	6.72	**TIC3**	6.40	**Mor31u**	7.51	**RTe**	7.08	nO	7.64	**nDB**	6.13
**BLTD4**	6.52	TIC5	6.22	GATS1m	7.05	MWC05	7.05	EEig10d	7.46	**MWC04**	6.05
**BELe3**	6.51	**Mor01m**	6.22	**EEig10r**	6.36	GNar	6.88	**J**	7.25	Mor23u	5.44
**BLTA96**	6.48	**Lop**	5.92	**MATS4p**	6.24	ATS1p	6.30	**ESpm14r**	7.12	Mor23e	5.28
**Mor08e**	5.52	ESpm11x	5.49	ESpm05u	6.11	**ATS3m**	6.26	**MWC04**	6.61	**ESpm14r**	5.21
**Mor10p**	5.29	**GATS2e**	5.26	HATS3u	6.09	**ESpm14d**	6.25	**ESpm01d**	6.50	**ESpm13d**	4.77
**Mor15p**	5.29	**Mor31e**	5.22	Jhetm	5.75	**MLOGP**	5.83	**nDB**	6.41	RDF040m	4.67
**MLOGP**	5.11	VRv1	5.17	ADDD	5.33	**GATS2e**	5.78	H0p	6.20	GGI2	4.65
**Lop**	5.09	**Mor31u**	4.96	**MLOGP**	5.31	MATS6p	5.23	**ESpm07d**	6.16	SPAN	4.65
**Mor10v**	5.05	ATS2p	4.95	**GATS2p**	5.23	**MATS7v**	5.21	AMW	5.93	**ATS3m**	4.62
**Mor31e**	4.98	**Mor24u**	4.95	**Mor15p**	5.08	ATS1m	5.19	X1sol	5.90	**RARS**	4.61
**Mor15v**	4.87	L3e	4.92	Mor07u	4.95	**RARS**	4.91	AECC	5.79	EEig09d	4.61
**VRp2**	4.74	ICR	4.82	**GATS2v**	4.92	**Mor24e**	4.85	**Mor02p**	5.73	QXXv	4.58
**VRv2**	4.74	ESpm08u	4.80	MATS6e	4.78	**GATS2m**	4.78	**Ui**	5.68	Mor21u	4.21
**Mor31u**	4.63	ESpm10x	4.72	**GATS2m**	4.77	CIC1	4.78	HDcpx	5.08	**ALOGP**	4.17
Mor03u	4.60	**Mor08e**	4.64	**RBN**	4.75	L2e	4.67	**ESpm13d**	5.03	EEig10x	4.12

Note: Bold format is used to highlight those MDs appearing in more than one chromatographic system.

**Table 3:** HILIC column specifications and chromatographic separation conditions used during the analysis.

Column Specifications				Chromatographic Separation Conditions	
Name	Manufacturer	Stationary Phase	Dimensions	Flow (mL·min^−1^)	Elution Gradient (A: Acetonitrile; B: Water with Ammonium Acetate)
XBridge^TM^ Amide	Waters (Milford, MA, USA)	BEH amide	150 × 4.6 mm^2^ i.d., 5 μm	0.15	0–4 min, at 5% B; 4–34 min, from 5% to 70% B; 34–42 min, at 70% B; and 42–44 min, at 5%B
TSK Gel Amide-80	Tosoh Bioscience (Tokyo, Japan)	Amide	250 × 2.0 mm^2^ i.d., 5 μm	0.15	0–3 min, at 5% B; 3–27 min, from 5% to 70% B; 27–30 min, at 70% B; and 30–32 min, at 5%B
ZIC-HILIC	SeQuant (Umeå, Sweden)	Zwitterionic	250 × 2.1 mm^2^ i.d., 5 μm	0.15	0–3 min, at 5% B; 3–27 min, from 5% to 70% B; 27–30 min, at 70% B; and 30–32 min, at 5%B
Acclaim^TM^ Mixed-Mode HILIC-1	Thermo Scientific (Sunnyvale, CA, USA)	Mixed-mode diol	150 × 2.1 mm^2^ i.d., 5 μm	0.15	0–2 min, at 5% B; 2–16 min, from 5% to 70% B; 16–20 min, at 70% B; and 20–22 min, at 5%B

## Supplementary Materials

The following are available online at www.mdpi.com/2218-1989/7/4/54/s1, Figure S1: PCA scores plot of samples classified according to the pH of the mobile phase. Table S1: Metabolites’ logP values obtained from PCLIENT software.
