# Supplementary material for: Modelling of Hydrophilic Interaction Liquid Chromatography Stationary Phases Using Chemometric Approaches
Source: Metabolites. 2017 Oct 24;7(4):54. doi: 10.3390/metabo7040054 (PMC5746734; doi:10.3390/metabo7040054)
Supplement: Supplementary file 1 [file metabolites-07-00054-s001.pdf]

Supplementary Information: Modeling of hydrophilic interaction liquid chromatography stationary phases using chemometric approaches

Meritxell Navarro-Reig [1]. Elena Ortiz-Villanueva [1]. Romà Tauler [1] and Joaquim Jaumot [1,*]

[1] Department of Environmental Chemistry. IDAEA-CSIC. Jordi Girona 18-26. 08034 Barcelona. Spain

* Correspondence: joaquim.jaumot@idaea.csic.es; Tel.: +34934006100-1443

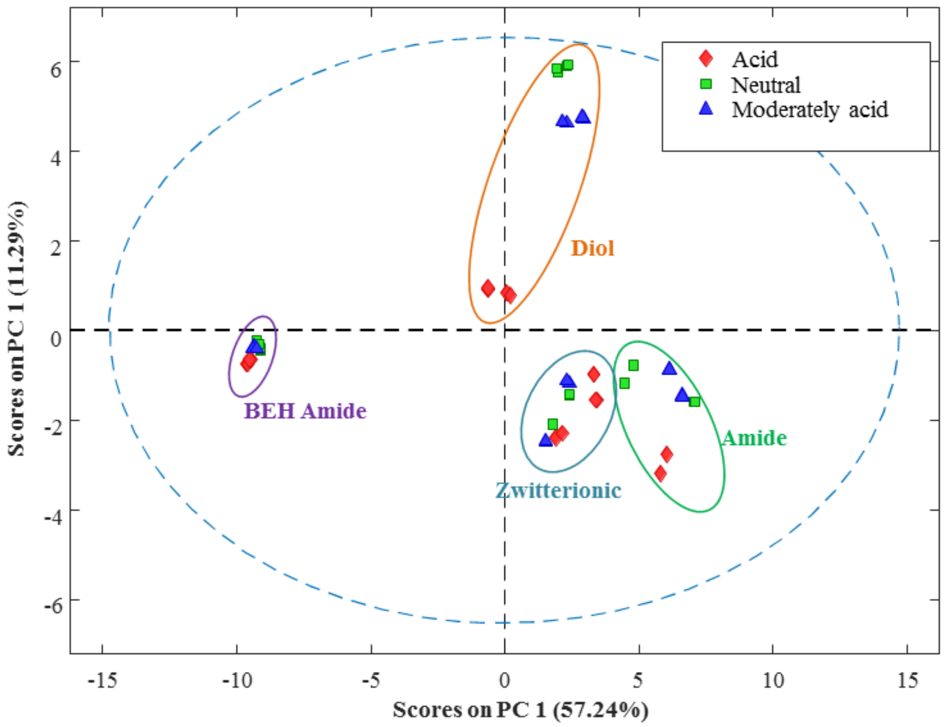

Figure S1.PCA scores plot of samples classified according to the pH of the mobile phase.

Table S1. Metabolites' logP values obtained from PCLIENT software.

**Metabolites**	**logP**
**Nucleosides**	
1-methyladenosine	−0.054
2''-O-methylcytidine	−0.872
2-thiocytidine	−0.427
5-methylcytidine	−0.872
cytidine	−1.176
guanosine	−0.808
inosine	−0.752

pseudouridine	−1.988
ribothymidine	−1.278
uridine	−1.582
**Amino Acids**	
1-methyl-L-histidine	−2.704
3-methyl-L-histidine	−2.704
4-hydroxy-L-proline	−1.04
5-hydroxylysine	−3.273
β-alanine	−2.918
creatinine	−0.339
cysteine	−5.555
L-(-)-proline	−0.232
L-(+)-arginine	−2.934
L-(+)-cystathionine	−5.202
L-(+)-lysine	−2.485
L-2-aminoadipic acid	−2.568
L-2-amino-n-butyric acid	−2.465
L-alanine	−2.918
L-anserine	−0.75
L-carnosine	−1.054
L-citrulline	−3.34
L-glutamic acid	−2.946
L-histidine	−3.057
L-homocystine	−4.869
L-isoleucine	−1.677
L-leucine	−1.677
L-methionine	−2.055
L-ornithine	−2.863
L-serine	−3.726
L-threonine	−3.272
L-tryptophan	−1.267
L-valine	−2.055
taurine	−1.731
sarcosine	−0.703
L-aspartic acid	−3.356
**Sugars**	
D(-)-ribose	−2.089
glucose	−2.483
trehalose	−3.898
mannitol	−2.497
**Organic acids**	
citric acid	−1.169
ketoglutaric acid	−0.828
pimelic acid	0.787
succinic acid	−0.353

creatine	-0.735
**Others**	
hypoxanthine	0.582
L-carnitine	−3.601
serotonin	1.006
tryptamine	1.574
